# Natural and induced growth of VO_2_ (M) on VO_2_ (B) ultrathin films

**DOI:** 10.1038/s41598-018-25656-6

**Published:** 2018-05-08

**Authors:** Nicolas Émond, Badr Torriss, Mohamed Chaker

**Affiliations:** INRS-Énergie, Matériaux et Télécommunications, 1650, Boulevard Lionel Boulet, Varennes, Québec, J3X 1S2 Canada

## Abstract

This work examines the synthesis of single phase VO_2_ (B) thin films on LaAlO_3_ (100) substrates, and the naturally-occurring and induced subsequent growth of VO_2_ (M) phase on VO_2_ (B) films. First, the thickness (*t*) dependence of structural, morphological and electrical properties of VO_2_ films is investigated, evidencing that the growth of VO_2_ (B) phase is progressively replaced by that of VO_2_ (M) when *t* > ~11 nm. This change originates from the relaxation of the substrate-induced strain in the VO_2_ (B) films, as corroborated by the simultaneous increase of surface roughness and decrease of the c-axis lattice parameter towards that of bulk VO_2_ (B) for such films, yielding a complex mixed-phase structure composed of VO_2_ (B)/VO_2_ (M) phases, accompanied by the emergence of the VO_2_ (M) insulator-to-metal phase transition. Second, the possibility of inducing this phase conversion, through a proper surface modification of the VO_2_ (B) films via plasma treatment, is demonstrated. These natural and induced VO_2_ (M) growths not only provide substantial insights into the competing nature of phases in the complex VO_2_ polymorphs system, but can also be further exploited to synthesize VO_2_ (M)/VO_2_ (B) heterostructures at the micro/nanoscale for advanced electronics and energy applications.

## Introduction

Vanadium dioxide (VO_2_) is a particularly interesting polymorphic material family that exists under several forms including VO_2_ (A), VO_2_ (B), VO_2_ (M_1_), VO_2_ (M_2_) and VO_2_ (M_3_)^1^. Even though the chemical formula remains the same, VO_2_ can assume various crystalline symmetries and electronic structures that exhibit different electronic and optical properties on account of strong correlation^2,3^. This diversity of polymorphs makes VO_2_ a promising electronic material and an excellent candidate for technological applications such as batteries, optical and electronic switching devices, IR sensors, smart windows and tunable metamaterials^4–13^. VO_2_ (M) and VO_2_ (B) are the most desired VO_2_ polymorphs as they display large changes in their electrical resistivity with temperature.

VO_2_ (M) is stable at room temperature and exhibits a monoclinic structure with the *P2*_1_*/c* (14) space group and lattice parameters *a*_M_ = 5.75 Å, *b*_M_ = 4.54 Å, *c*_M_ = 5.38 Å and *β* = 122.6°^14–16^. It undergoes a structural first-order reversible insulator-to-metal transition (IMT) at a critical temperature of *T*_IMT_ ≈ 68 °C to a VO_2_ (R) rutile structure with the *P4*_2_*/mmm* (136) space group and corresponding lattice parameters *a*_R_ = *b*_R_ = 4.55 Å and *c*_R_ = 2.85 Å. This transition is accompanied by sharp changes of both electrical resistivity and infrared reflectivity. The VO_2_ (M) phase is characterized by V-V dimerization, and alternatively short (~2.65 Å) and long (~3.12 Å) V-V bonds that result in localization of d orbital electrons to individual ions, which yields an insulating material. In the VO_2_ (R) phase, all the V-V bonds are equidistant (~2.87 Å), so that the d orbital electrons are shared by all the vanadium ions along the V-V chain, which leads to a metallic behavior^17^.

The VO_2_ (B) metastable phase assumes a monoclinic layered structure similar to that of V_6_O_13_ with the *C2/m* (12) space group and lattice parameters *a*_B_ = 12.03 Å, *b*_B_ = 3.69 Å, *c*_B_ = 6.42 Å and *β* = 106.6°^18^. Unlike VO_2_ (M), the decrease of the VO_2_ (B) electrical resistivity across the phase transition occurs gradually over a very broad range of temperatures, decaying by ~4 orders of magnitude when heating from −123 °C to 127 °C. This transition is characterized by a change in the structure from a monoclinic semiconducting phase to another monoclinic semi-metallic phase^1^. While the layered structure is maintained across the transition, the distance between neighboring V^4+^ ions slightly decreases, the reduction being larger for the ions in the (*ac*) plane, which represent half of the cations in the VO_2_ (B) structure.

Recent experiments have demonstrated the possibility to synthesize textured VO_2_ (B) thin films via pulsed laser deposition by lattice matching with SrTiO_3_ (001) and LaAlO_3_ (100) substrates^10,19^. These results indicate that the thinnest VO_2_ (B) films do not contain any VO_2_ (M) phase and that the fraction of VO_2_ (M) phase increases with the film thickness. However, the mechanism that governs the emergence of the VO_2_ (M) phase and its distribution among the VO_2_ (B) phase remains unclear. Besides their individual properties, combining the properties of the two phases by co-growing these VO_2_ polymorphs or by designing and fabricating VO_2_ (M)/VO_2_ (B) heterostructures could be exploited for future optoelectronic materials with tunable properties suitable in advanced electronic and energy devices.

Herein, we first investigate the structural, morphological and electrical properties of VO_2_ thin films of various thickness (3 nm ≤ *t* ≤ 38 nm) grown on LaAlO_3_ (100) (LAO) substrates. We demonstrate that the growth of distorded monoclinic VO_2_ (B) metastable phase breaks at a critical thickness (*t*_c_) between 11 and 25 nm. Beyond that critical thickness, the strain-induced structural change further promotes the growth of the VO_2_ (M) phase, which in turn results in the presence of a complex mixed-phase structure composed of VO_2_ (B) and VO_2_ (M) and in the appearance of the VO_2_ (M) insulator-to-metal phase transition. We further exploit this behavior and demonstrate the possibility to tailor the growth of VO_2_ polymorphs by modifying the properties of an ultrathin VO_2_ (B) film via treatment of the film surface with an argon plasma. This process significantly modifies the vanadium valence state at the VO_2_ (B) film surface and further promotes the growth of the VO_2_ (M) phase.

## Results

### Natural growth of VO_2_ (M) on VO_2_ (B)

**Figure 1:**
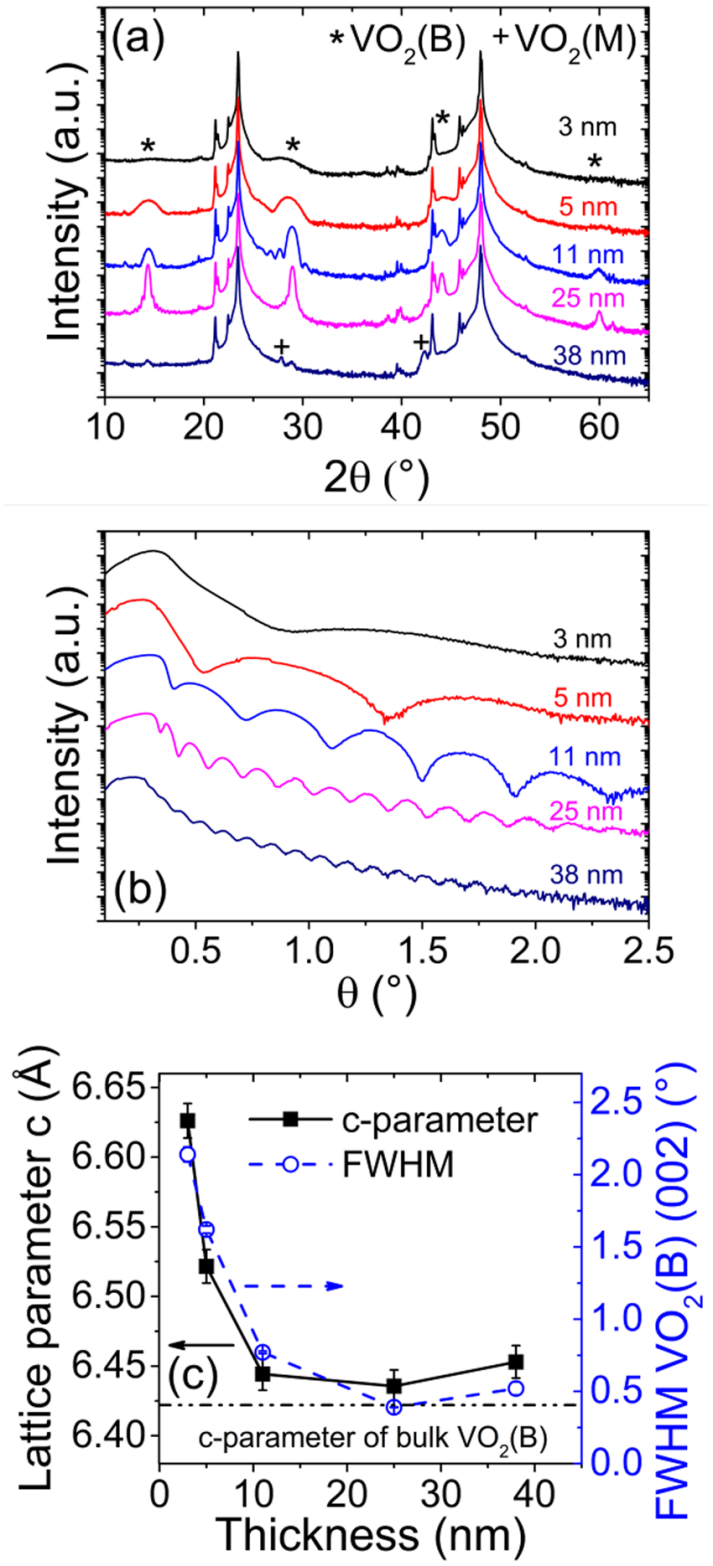
Thickness dependent structural properties of VO_2_ films. (**a**) X-ray diffraction θ-2θ patterns, (**b**) XRR curves and (**c**) lattice parameter c and FWHM of the VO_2_ (B) (002) peak of VO_2_ thin films grown on LAO substrates as a function of film thickness ranging from 3 to 38 nm.

The quality of the VO_2_/LAO thin films was evaluated by acquiring XRD diffraction patterns of films with thicknesses ranging from 3 to 38 nm, as shown in Fig. 1(a). An interesting modification of the structure with thickness is observed. A detailed analysis reveals the presence of VO_2_ (B) (00 *l*) peaks for film thickness between 3 and 25 nm, which suggests that the films are highly oriented along this direction. However, the thickest film (38 nm) behaves differently, with the intensity of the VO_2_ (B) (00 *l*) peaks being significantly smaller, while two other peaks appear at 2θ = 27.8° and 42.2°. These angles correspond to the Bragg angle of the (011) and (210) orientations of the VO_2_ (M) phase, respectively. All the other peaks coincide with reflection planes from the LAO substrate. The lower intensity observed for the VO_2_ (M) (011) and (210) peaks in the diffractogram of the 38 nm-thick film as compared to those of the VO_2_ (B) (00 *l*) peaks in the diffractograms of the thinner films (3–25 nm) is due to the difference in both the diffracting planes Miller indices and crystalline structure between VO_2_ (M) and VO_2_ (B) phases. Accordingly, the structure factor (*F*) associated with each of these phases diffracting planes is different, and so is the diffracted intensity (*I* ∝ *F*^2^). For films with thickness below or equal to 25 nm, all peaks located at 2θ = 14.4°, 28.9°, 44 0° and 60.0° (corresponding to the Bragg angle of VO_2_ (B) (001), (002), (003) and (004) orientations respectively) show a full width at half maximum (FWHM) that decreases with increasing thickness, while their intensity increases and their position shifts to higher 2θ values.

The presence of well-defined thickness-interference (Kiessig) fringes in the X-ray reflectivity (XRR) curves of Fig. 1(b) indicates that the film surface is smooth and the film-substrate interface well defined^20^. The film thickness (*t*) can thus be calculated from these XRR curves from equation (1)^21^

1 $$t=\frac{(m-n)\lambda }{2(\sin \,{\theta }_{m}-\,\sin \,{\theta }_{n})}$$

Where *m* and *n* are the orders of interference, and *θ*_*m*_ and *θ*_*n*_ are the corresponding diffraction angles, respectively. The thickness values obtained from the XRR measurements are consistent with those determined from the cross-section SEM images of a VO_2_ test sample. Figure 1(c) shows the c-axis parameter and the FWHM of the (002) peak. The c-axis parameter gradually decays from 6.63 Å for the 3 nm-thick film to 6.44 Å for the 25 nm-thick film, which closely corresponds to the theoretical value of bulk VO_2_ (B) (*c*_B_ = 6.42 Å), while its FWHM decreases from 2.14 ° to 0.39°. The decrease of the c-axis parameter means that the strain in the VO_2_ films is gradually released as thickness grows.

**Figure 2:**
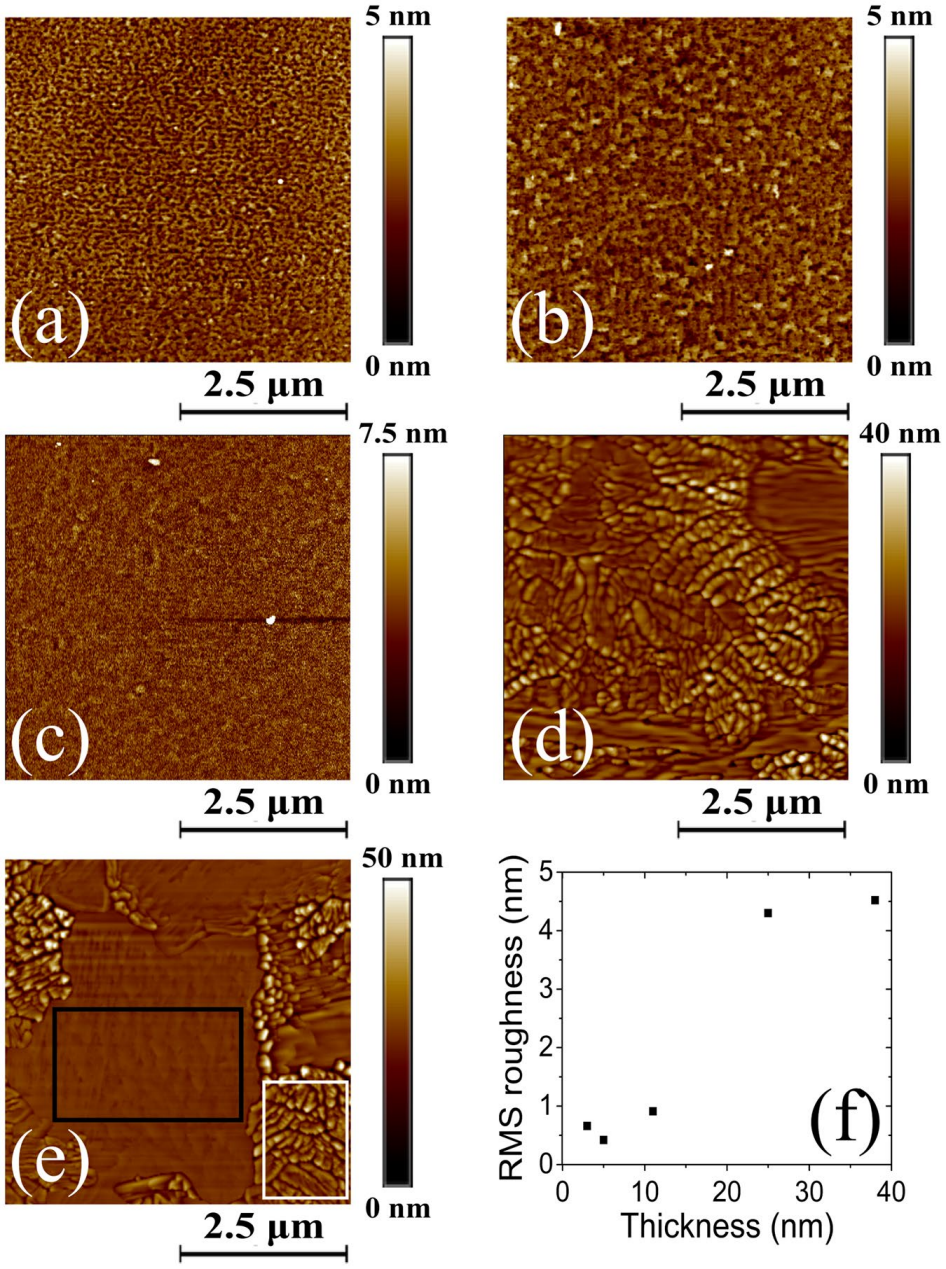
Thickness dependent morphological properties of VO_2_ films. AFM images of (**a**) 3, (**b**) 5, (**c**) 11, (**d**) 25 and (**e**) 38 nm-thick VO_2_ thin films grown on LAO substrates. (**f**) RMS surface roughness as a function of film thickness. The white and black rectangles in (**e**) represent the rough and flat regions, which have a large RMS roughness and a RMS roughness comparable to that of the thinnest VO_2_ films (**a–c**), respectively.

The observed thickness-dependent behavior of the VO_2_/LAO system can also be illustrated by observing the film surface topography. The AFM images of Fig. 2(a–e) indicate that the thinnest films (3 to 11 nm) are very smooth. On the other hand, the thicker films (25 and 38 nm) show the presence of some regions with granular morphology along with other regions where the surface remains flat. Accordingly, as depicted in Fig. 2(f), the RMS surface roughness of the films increases with thickness with a more drastic change (from ≈ 0.7 to ≈ 4.5 nm) between 11 and 25 nm. The presence of distinctive regions characterized by different morphologies on the surface of the 25 and 38 nm-thick films is evidenced in Fig. 2(d–e). More specifically, for the 38 nm-thick film (Fig. 2(e)), the measured RMS roughness increases from 0.81 nm for the flat region (black rectangle), in agreement with the measured values for the thinnest VO_2_ (B) films, to 6.94 nm for the rough region (white rectangle). Combining both AFM and XRD analysis strongly suggests that these rough regions are composed of VO_2_ (M) phase nanocrystallites that grow at the expense of the VO_2_ (B) phase.

**Figure 3:**
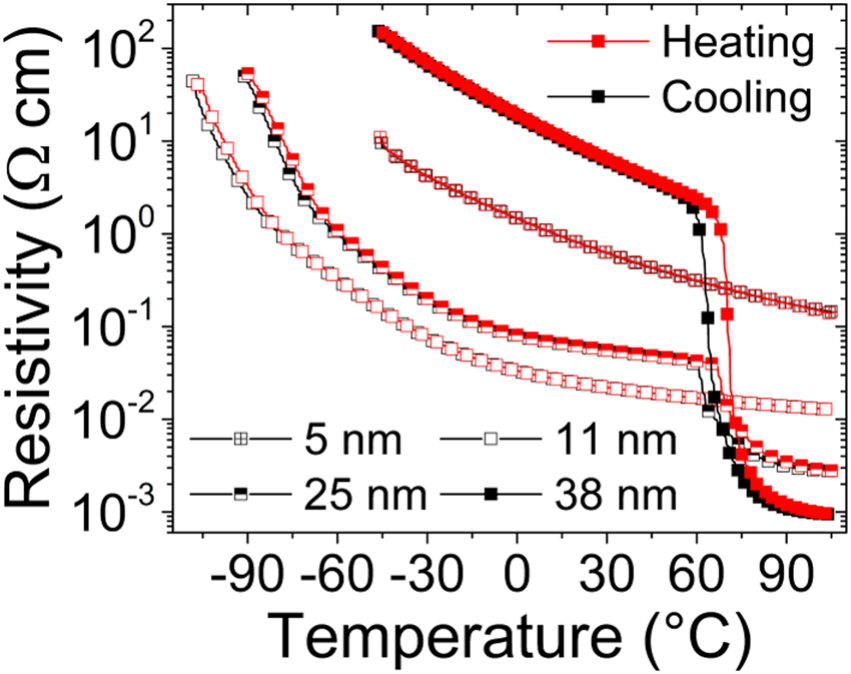
Thickness dependent electrical properties of VO_2_ films. Temperature-dependent resistivity of 5, 11, 25 and 38-nm thick VO_2_ thin films grown on LAO substrates for heating (red curve) and cooling (black curve) segments using a van der Pauw geometry.

Figure 3 shows the electrical resistivity of the VO_2_ films with different thicknesses as a function of the temperature for both heating and cooling. The resistivity of the 3 nm-thick film could not be measured as it was beyond the upper detection limit of the system over the available range of temperatures. Such behavior most likely originates from an early stage VO_2_ film growth that is driven by Volmer-Weber island-type mechanism^22,23^, which is characterized by the formation of unconnected separated islands. The film discontinuity therefore prevents the formation of a current pathway between the electrical contacts and rules out the possibility to reliably measure the 3 nm-thick VO_2_ film resistivity. The monotonous resistivity decrease with increasing temperature for the thinnest films (5 and 11 nm) is accompanied by a small hysteresis at low temperature, in agreement with the typical behavior of VO_2_ (B) thin films^24^. On the other hand, for the 38 nm-thick VO_2_ film, the temperature-dependent resistivity behaves like that of VO_2_ (M) phase^25^ as it decreases from 9.14 Ω cm at *T* = 20 °C in the insulating state to 0.96 mΩ cm at *T* = 105 °C in the metallic state. This provides evidence of the presence of the typical VO_2_ (M) insulator-to-metal transition with transition temperatures *T*_IMT(h)_ = 70.6 °C for the heating cycle and *T*_IMT(c)_ = 63.8 °C for the cooling cycle, as calculated from the d(log(ρ))/d*T* curves. The average transition temperature is therefore *T*_IMT_ = 67.2 °C, a value similar to that of bulk VO_2_ (M) (*T*_IMT_ ≈ 68 °C), while the hysteresis width is 6.8 °C. Finally, the 25 nm-thick film exhibits a monotonous decrease of the resistivity in the temperature range [−90 °C – 55 °C], in agreement with the VO_2_ (B) phase behavior. However, at *T* ≈ 65 °C, this film shows an abrupt decrease of its resistivity (from 40 mΩ cm to 6 mΩ cm), as observed for the VO_2_ (M) phase^14^.

Overall, the thickness dependence of the phase of the VO_2_ thin films deposited on LAO substrates clearly indicates that the exclusive growth of VO_2_ (B) ceases at some critical thickness where the growth of highly-oriented metastable monoclinic VO_2_ (B) films is no longer possible. Beyond that thickness, the monoclinic VO_2_ (M) phase appears and the films are then composed of a mixture of VO_2_ (B) and VO_2_ (M) polymorphs.

The VO_2_ (B) monoclinic structure observed for the thinnest VO_2_ thin films results from the good lattice matching and from the corresponding consistent strain with the LAO substrate. For films thicker than 11 nm, removing this strain through the creation of dislocations and defects strongly modifies locally and randomly the film surface morphology. Accordingly, the growth of the VO_2_ (B) polymorph is prevented, which promotes that of the VO_2_ (M) polymorph and leads to the coexistence of both VO_2_ (B) and VO_2_ (M) phases for films with thickness *t* > *t*_c_. These observations therefore indicate that strain significantly influence the competing nature of VO_2_ polymorphs growth, so as it does for the ultrafast structural transition dynamics following above-gap photoexcitation^26^. Likewise, they strongly suggest that the surface properties of the VO_2_ (B) film, which play a key role in the observed phase growth change from VO_2_ (B) to VO_2_ (M), should definitely be considered to further control the growth of these polymorphs. Indeed, a local modification of the surface of the VO_2_ (B) films, which is very smooth for the thinnest VO_2_ films, could locally be induced to promote the growth of the VO_2_ (M) phase in specific regions.

### Induced growth of VO_2_ (M) on VO_2_ (B) ultrathin films

To explore the possibility of inducing the growth of the VO_2_ (M) phase, a 5 nm-thick VO_2_/LAO sample displaying only the VO_2_ (B) phase was exposed to an argon plasma to modify its surface. In this experiment, performed at 20 °C, half of the sample surface was covered by a bare LAO substrate and the full sample was further exposed for 30 seconds to an argon plasma at a pressure of 10 mTorr with a substrate bias of 135 V. This experiment, which enables to expose only half of the ultrathin VO_2_ film as the other half remains as deposited, also ensures that the properties of the VO_2_ film surface are absolutely identical prior to the plasma treatment. After plasma treatment, the bare LAO substrate was removed and another 5 nm of VO_2_ was deposited over the whole sample. This yields on one half of the sample a 10 nm-thick untreated VO_2_ film and on the other half a mixed 10 nm-thick VO_2_ film composed of a 5 nm-thick treated layer covered with a 5 nm-thick untreated layer.

**Figure 4:**
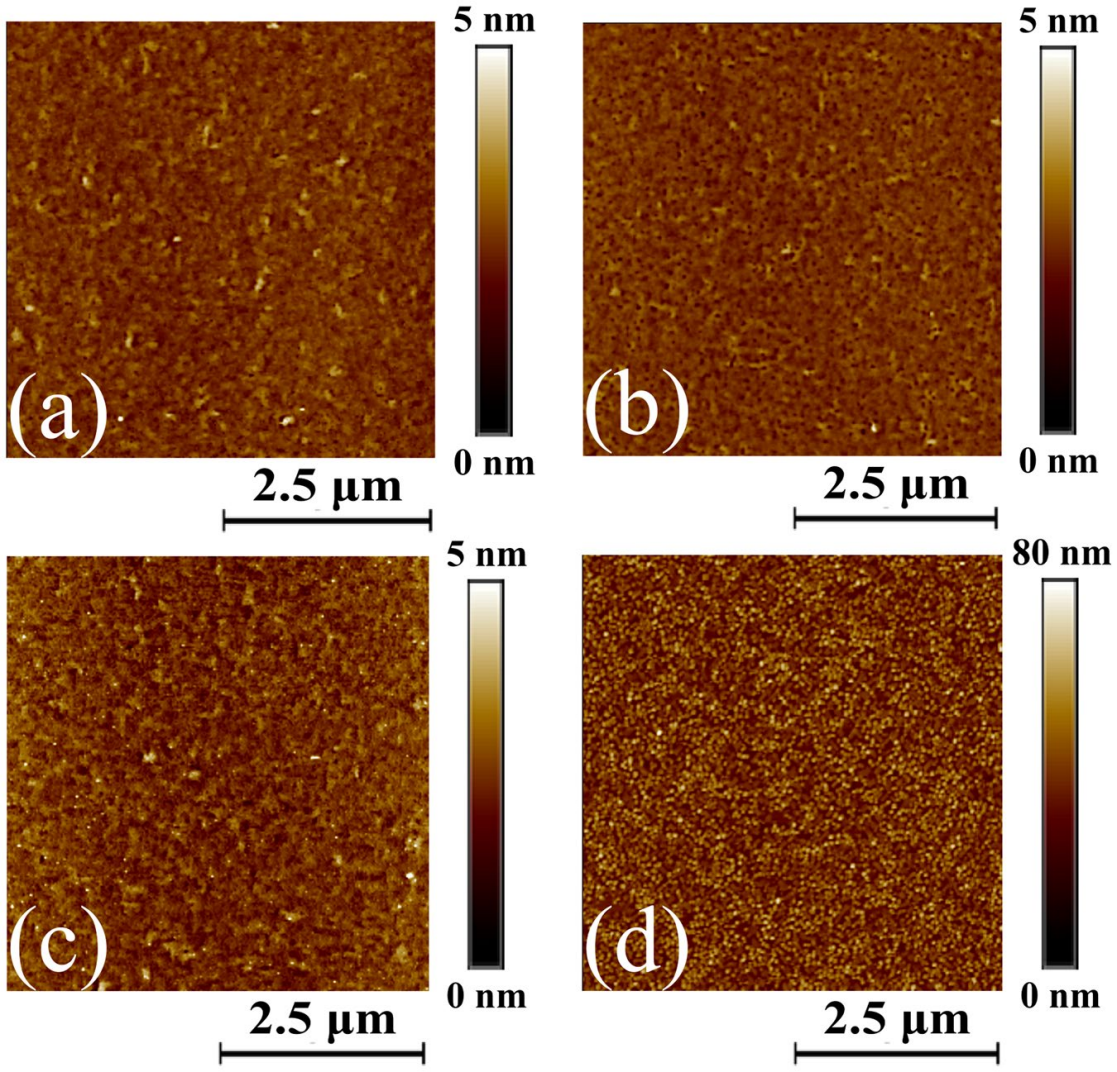
Effect of surface modification by plasma treatment on the morphological properties of the VO_2_ films. AFM images of the (**a**) untreated (5 nm UT) and (**b**) plasma-treated (5 nm T) 5 nm-thick VO_2_ thin films on LAO substrate and of the 5 nm-thick VO_2_ films deposited on (**c**) untreated (10 nm UT) and (**d**) plasma-treated (10 nm T) 5 nm-thick VO_2_ thin film surface.

**Figure 5:**
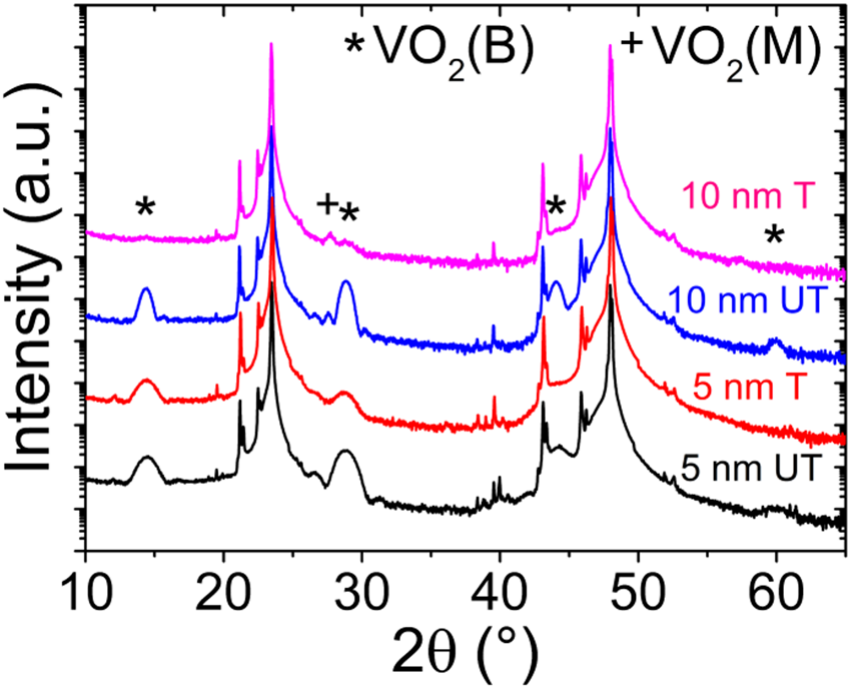
Effect of surface modification by plasma treatment on the structural properties of the VO_2_ films. X-ray diffraction θ-2θ patterns of the untreated (5 nm UT) and plasma-treated (5 nm T) 5 nm-thick VO_2_ thin films on LAO substrate and of the 5 nm-thick VO_2_ films deposited on the untreated (10 nm UT) and plasma-treated (10 nm T) 5 nm-thick VO_2_ thin film surface.

The morphological (AFM) and structural (XRD) properties of each half of the VO_2_ film were characterized prior to and after plasma treatment, and also after the second VO_2_ deposition. Figures 4 and 5 show the surface morphology and diffraction patterns of the untreated film (5 nm UT), of the treated film (5 nm T), of the film deposited on the untreated surface (10 nm UT) and of the film deposited on the plasma-treated surface (10 nm T). It is observed that plasma treatment modifies neither the morphology of the 5 nm-thick VO_2_ film nor its RMS surface roughness. Indeed, the topography of both 5 nm UT (Fig. 4(a)) and 5 nm T (Fig. 4(b)) is flat and smooth with small RMS roughness values of 0.32 nm and 0.29 nm, respectively. The structural properties are also unaffected by the plasma treatment as the diffractograms of both 5 nm UT and 5 nm T films exclusively display VO_2_ (B) (00 *l*) peaks. Nevertheless, a slight intensity reduction is observed for the VO_2_ (B) (00 *l*) peaks present in the diffractogram of the 5 nm T film with regards to that of the 5 nm UT film. This small intensity reduction could result from a plasma treatment-induced surface amorphization within the top first few atomic layers of the 5 nm T film. While the surface morphology of the 10 nm UT film (Fig. 4(c)) remains similar to that of the 5 nm UT and the 5 nm T films with only a slightly higher RMS roughness of 0.42 nm, that of the 10 nm T film (Fig. 4(d)) is significantly modified. In this case, the surface is covered with nanograins and its RMS roughness increases to 9.86 nm. In addition, the structural properties of the 10 nm T film are significantly altered as compared to those of the 10 nm UT film. Indeed, as shown in Fig. 5, the intensity of the VO_2_ (B) (00 *l*) peaks of the 10 nm T film significantly decreases as compared with that of the three other films while the VO_2_ (M) (011) peak emerges. Clearly, these effects exclusively result from the plasma treatment of the underlying VO_2_ ultrathin film, which indicates that this treatment has induced a modification of the growth mechanisms. Interestingly, these effects are also similar to those previously observed when the thickness of the VO_2_ films is increased from 11 nm to 38 nm so as to exceed the critical thickness.

### Origin of the induced growth

**Figure 6:**
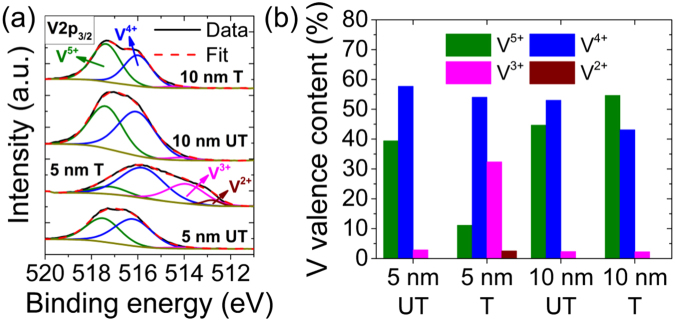
Effect of surface modification by plasma treatment on the vanadium valence state content of the VO_2_ films. (**a**) XPS spectra of the V2p_3/2_ peak deconvoluted into V^5+^, V^4+^, V^3+^ and V^2+^ peaks and (**b**) vanadium valence content of the untreated (5 nm UT) and plasma-treated (5 nm T) 5 nm thick VO_2_ thin films on LAO substrate and of the 5 nm-thick VO_2_ films deposited on the untreated (10 nm UT) and plasma-treated (10 nm T) 5 nm-thick VO_2_ thin film surface.

To further gain insight in the mechanisms governing the phase growth change induced by plasma treatment, X-ray photoelectron spectroscopy (XPS) was performed on the four samples (5 nm UT, 5 nm T, 10 nm UT and 10 nm T). The V2p_3*/*2_ core level binding energy was used to characterize the oxidation state of vanadium^27^. Therefore, the vanadium valence state content of the VO_2_ films was determined at each step of the process by deconvoluting the V2p_3*/*2_ peak in a combination of V^5+^, V^4+^, V^3+^ and V^2+^ Gaussian/Lorentzian peaks, as depicted in Fig. 6(a). The position of these peaks was ascribed to 517.4 eV, 516.1 eV, 514.1 eV and 512.9 eV, respectively^28^ and the spectra were fitted using the CasaXPS software and a Shirley function to remove the background. The vanadium valence state content of the VO_2_ films shown in Fig. 6(b) was calculated from the ratio of the integrated areas of the V^5+^, V^4+^ V^3+^ and V^2+^ peaks.

The spectrum of the 5 nm UT film is mainly due to the V^5+^ (39.4%) and V^4+^ (57.7%) valence states with negligible contribution from V^3+^ (2.9%) and none from V^2+^ valence states. It is similar to that of the 10 nm UT film, but strongly differs from that of the 5 nm T film. Indeed, the latter film shows a broader XPS spectrum that is shifted to lower binding energies, so that the contribution from the V^5+^ valence state is strongly reduced to 11.1% and that of the V^3+^ valence state is enhanced from 2.9% to 32.4%, while a small contribution from the V^2+^ valence state (2.5%) appears. For the 10 nm T film, the spectrum width shrinks back and returns to higher binding energies, like 5 nm UT and 10 nm UT films. Accordingly, the main contributions comes from the V^5+^ (54.6%) and V^4+^ (43.1%) valence states while the V^3+^ valence state negligibly contributes (2.5%) and the V^2+^ not at all. One can therefore conclude that plasma treatment strongly modifies the surface chemical states of the ultrathin VO_2_ (B) film, lowering the contribution of the higher valence state (V^5+^) and enhancing that of the lower valence states (V^3+^ and V^2+^). This indicates a larger amount of oxygen vacancies at the surface^29–31^, which favors the growth of the VO_2_ (M) phase as compared to that of VO_2_ (B). Furthermore, even though the stoichiometry and chemical states of the VO_2_ (B) film are significantly modified by plasma treatment, the stoichiometry of the subsequently grown VO_2_ film is preserved.

The change of phase growth induced by plasma treatment strongly affects the structural and morphological properties of the VO_2_ ultrathin film. Indeed, the 2D growth of pseudomorphic metastable VO_2_ (B) film exhibiting a flat surface topography switches to a 3D island-type growth of stable VO_2_ (M) nanocrystallites with rough surface, forming a VO_2_ (B)/VO_2_ (M) composite structure. These effects are very similar to those observed when the VO_2_ film thickness is increased beyond the critical thickness lying between 11 and 25 nm. However, tailoring the phase growth offers the possibility to spatially control both the size and location of the emerging VO_2_ (M) phase regions using for example a patterning method such as lithography. In contrast, natural phase growth results in randomly distributed phase mixtures. Tailoring the phase growth could thus be exploited to control the VO_2_ structure at the nanometer scale through the onset of spatially controlled VO_2_ (M) islands in a VO_2_ (B) ultrathin film, paving the way to the synthesis of various VO_2_ (M)/VO_2_ (B) complex heterostructures.

## Conclusions

In conclusion, by investigating the morphological, structural and electrical properties of VO_2_ films grown on LAO substrates with various thickness (3 nm ≤ *t* ≤ 38 nm), the growth of single pseudomorphic distorted metastable monoclinic VO_2_ (B) phase was shown to change once a critical thickness lying between 11 and 25 nm is reached. This strain-induced change of structural phase growth is accompanied by the increase of surface roughness and by the appearance of the stable VO_2_ (M) phase. The thicker films exhibit a complex mixed-phase structure composed of VO_2_ (B) and VO_2_ (M) polymorphs and undergo the typical VO_2_ (M) first-order phase transition at *T*_IMT_ ≈ 68 °C. By modifying the VO_2_ (B) ultrathin film using plasma treatment, the possibility to induce this change of phase growth was demonstrated and further related to a strong modification of the vanadium valence state on the VO_2_ (B) film surface and to the corresponding creation of oxygen vacancies. Natural or induced phase growth change not only provides a stimulating environment for investigating the fundamental issues related to the complex competing nature of the VO_2_ polymorphs, but also presents a strong potential for the fabrication of VO_2_ (M)/VO_2_ (B) heterostructures at the nanoscale. This opens new opportunities of applications in the field of advanced electronics and energy where nanostructured electronic materials with tunable properties are required, as well as for the design of metamaterials for optoelectronic applications.

## Methods

### Sample growth

Reactive pulsed laser deposition (RPLD) was used to fabricate VO_2_ thin films by ablating a commercial vanadium metal target (99.95% purity, KJ Lesker). The films were deposited on LaAlO_3_ (100) substrates. The growth temperature was 550 °C and the oxygen pressure was kept at 21 mTorr. The detailed growth conditions were reported in a previous study^10^.

### XRD, AFM, XPS and electrical characterization

The structural properties of the films were examined by X-ray diffraction (XRD) in the θ–2θ configuration, between 10° and 65°, and in the X-ray reflectivity (XRR) configuration using a PANalytical’s X’Pert PRO Materials Research Diffractometer with Cu Kα radiation operated at 45 kV and 40 mA. The film thickness was determined by cross-section scanning electron microscopy (SEM, JEOL JSM-7401F) on a test sample and by X-ray reflectivity. The surface morphology of the films was imaged by atomic force microscopy (AFM, DI-EnviroScope, Veeco) while X-ray photoelectron spectroscopy (XPS) measurements were carried out using a VG Escalab 220I-XL system with Al Kα (hν = 1486.6 eV) radiation. The resistivity of the films was measured in van der Pauw geometry using a Quantum Design physical properties measurement system (PPMS) and a cryostat. The electrical contacts (100 nm Au/300 nm Cu/5 nm Cr) were deposited at the corners of the samples using e-beam evaporation.

### Surface treatment by argon ions

Surface treatment was achieved in a cylindrical Inductively Coupled Plasma reactor (ICP) from Oxford instruments (Plasmalab 100, model ICP 380). In this system, the ICP plasma was generated at a frequency of 2 MHz and the power was set at 1 kW. The kinetic energy of the ions was controlled by applying 13.56 MHz RF power on the chuck table, yielding a bias voltage of 135 V. The experiments were carried out in pure Ar and the gas pressure (10 mTorr) was controlled by means of a throttling valve located at the bottom of the processing chamber.

### Data availability

The data that support the findings of this study are available from the corresponding author on request.

## References

[1] Srivastava A (2015). Selective growth of single phase VO_2_(A, B, and M) polymorph thin films. APL Mater..

[2] Imada M, Fujimori A, Tokura Y (1998). Metal-insulator transitions. Rev. Mod. Phys..

[3] Basov DN, Averitt RD, van der Marel D, Dressel M, Haule K (2011). Electrodynamics of correlated electron materials. Rev. Mod. Phys..

[4] Mai L (2013). Nanoscroll buffered hybrid nanostructural VO_2_(B) cathodes for high-rate and long-life lithium storage. Adv. Mater..

[5] Pei C (2017). VO_2_ nanoflakes as the cathode material of hybrid magnesium−lithium-ion batteries with high energy density. ACS Appl. Mater. Interfaces.

[6] Wu C, Wei H, Ning B, Xie Y (2010). New vanadium oxide nanostructures: Controlled synthesis and their smart electrical switching properties. Adv. Mater..

[7] Appavoo K (2014). Ultrafast phase transition via catastrophic phonon collapse driven by plasmonic hot-electron injection. Nano Lett..

[8] Shukla N (2015). A steep-slope transistor based on abrupt electronic phase transition. Nat. Commun..

[9] Zhi B (2014). Electric-field-modulated nonvolatile resistance switching in VO_2_/PMN-PT(111) heterostructures. ACS Appl. Mater. Interfaces.

[10] Émond N, Hendaoui A, Chaker M (2015). Low resistivity W_x_V_1−x_O_2_-based multilayer structure with high temperature coefficient of resistance for microbolometer applications. Appl. Phys. Lett..

[11] Zhou J (2013). VO_2_ thermochromic smart window for energy savings and generation. Sci. Rep..

[12] Chen Z (2016). Self-assembled, nanostructured, tunable metamaterials via spinodal decomposition. ACS Nano.

[13] Émond N, Torriss B, Morris D, Chaker M (2017). Natural metamaterial behavior across the phase transition for W_x_V_1-x_O_2_ films revealed by terahertz spectroscopy. Acta Mater..

[14] Morin FJ (1959). Oxides which show a metal-to-insulator transition at the Neel temperature. Phys. Rev. Lett..

[15] Zylberstejn A, Mott NF (1975). Metal-insulator transition in vanadium dioxide. Phys. Rev. B.

[16] Eyert V (2002). The metal-insulator transitions of VO_2_: A band theoretical approach. Ann. Phys..

[17] Goodenough JB (1971). The two components of the crystallographic transition in VO_2_. J. Solid State Chem..

[18] Oka Y, Yao T, Yamamoto N, Ueda Y, Hayashi A (1993). Phase transition and V^4+^- V^4+^ pairing in VO_2_(B). J. Solid State Chem..

[19] Chen A (2014). Textured metastable VO_2_(B) thin films on SrTiO_3_ substrates with significantly enhanced conductivity. Appl. Phys. Lett..

[20] Sinha SK (1994). X-ray diffuse scattering as a probe for thin film and interface structure. J. Phys. III France.

[21] Lu Y (2000). Magnetoresistance of coherently strained La_2/3_Ba_1/3_MnO_3_/SrTiO_3_ superlattices. Phys. Rev. B.

[22] Paik H (2015). Transport properties of ultra-thin VO_2_ films on (001) TiO_2_ grown by reactive molecular-beam epitaxy. Appl. Phys. Lett..

[23] Rúa A, Díaz RD, Lysenko S, Fernández FE (2015). Semiconductor-insulator transition in VO_2_ (B) thin films grown by pulsed laser deposition. J. Appl. Phys..

[24] Corr SA (2009). VO_2_(B) nanorods: Solvothermal preparation, electrical properties, and conversion to rutile VO_2_ and V_2_O_3_. J. Mater. Chem..

[25] Nakano M (2012). Collective bulk carrier delocalization driven by electrostatic surface charge accumulation. Nature.

[26] He X (2017). Photoinduced strain release and phase transition dynamics of solid-supported ultrathin vanadium dioxide. Sci. Rep..

[27] Silversmit G, Depla D, Poelman H, Marin GB, De Gryse R (2004). Determination of the V2p XPS binding energies for different vanadium oxidation states (V^5+^ to V^0+^). J. Electron Spectrosc. Relat. Phenom..

[28] Hryha E, Rutqvist E, Nyborg L (2012). Stoichiometric vanadium oxides studied by XPS. Surf. Interface Anal..

[29] Xu HY (2016). Effects of annealing ambient on oxygen vacancies and phase transition temperature of VO_2_ thin films. RSC Adv..

[30] Zhang, J. *et al*. Evolution of structural and electrical properties of oxygen-deficient VO_2_ under low temperature heating process. *ACS Appl. Mater. Interfaces* **9**, 27135–27141 (2017). 10.1021/acsami.7b05792 28753266

[31] Jeong J (2013). Suppression of metal-insulator transition in VO_2_ by electric field-induced oxygen vacancy formation. Science.

